# Regulatory B cells are induced in untreated HIV-1 infection and suppress HIV-1 specific T cell responses

**DOI:** 10.1186/1742-4690-9-S2-P102

**Published:** 2012-09-13

**Authors:** J Liu, W Zhan, C Kim, E Lee, J Cao, B Ziegler, A Gregor, F Yue, S Huibner, S Macparland, K Clayton, J Schwartz, H Song, E Bento, C Kovacs, R Kaul, M Ostrowski

**Affiliations:** 1 University of Toronto, Toronto, Canada; 2 Maple Leaf Clinic, Toronto, Canada

## Background

Regulatory B cells (Breg), the B cells producing interleukin 10 (IL-10), have been identified in mice and humans. Mouse Breg can suppress innate and T cell responses and are implicated in pathogenesis of some autoimmune diseases and immune evasion of some pathogens. However, the role of Breg in humans is less clear.

## Methods

PBMC and gut biopsy samples were obtained from healthy donors and HIV infected individuals. Flow cytometry and Luminex were used to quantify cytokine production. Flow cytometry were used to analyze Breg's phenotype.

## Results

Breg were elevated in both peripheral blood and gut tissue of untreated HIV-1 infected individuals and the elevation correlated with viral load in early HIV-1 infection. Breg from HIV-1 infected individuals were CD19^+^TIM-1^+^.Anti-retroviral therapy could reduce elevated Breg frequency. Treatment of B cells from healthy donors with microbial translocation products could differentiate them toward a Breg phenotype. Ex vivo Bregs from HIV-1 infected individuals suppressed cytokine production /degranulation of HIV-1 specific T cells that was in part IL-10 dependent.

## Conclusion

Our findings show that Bregs are induced early in HIV-1 infection, which may play a role in inhibiting effective HIV-1-specific T cell responses.

